# *Claudin11* Promoter Hypermethylation Is Frequent in Malignant Melanoma of the Skin, but Uncommon in Nevus Cell Nevi

**DOI:** 10.3390/cancers7030834

**Published:** 2015-07-07

**Authors:** Sara K. Walesch, Antje M. Richter, Peter Helmbold, Reinhard H. Dammann

**Affiliations:** 1 Institute for Genetics, Justus-Liebig-University Giessen, D-35392 Giessen, Germany; E-Mails: sara.walesch@gen.bio.uni-giessen.de (S.K.W.); antje.richter@gen.bio.uni-giessen.de (A.M.R.); 2 Department of Dermatology, University of Heidelberg, D-69120 Heidelberg, Germany; E-Mail: peter.helmbold@med.uni-heidelberg.de

**Keywords:** malignant melanoma, *Claudin 11*, tumor suppressor gene, epigenetics, DNA methylation

## Abstract

Epigenetic inactivation of tumor-related genes is an important characteristic in the pathology of human cancers, including melanomagenesis. We analyzed the epigenetic inactivation of *Claudin 11* (*CLDN11*) in malignant melanoma (MM) of the skin, including six melanoma cell lines, 39 primary melanoma, 41 metastases of MM and 52 nevus cell nevi (NCN). *CLDN11* promoter hypermethylation was found in 19 out of 39 (49%) of the primary MM and in 21 out of 41 (51%) of the MM metastases, but only in eight out of 52 (15%) of NCN (*p =* 0.001 and *p =* 0.0003, respectively). Moreover, a significant increase in the methylation level of *CLDN11* from primary melanomas to MM metastases was revealed (*p =* 0.003). Methylation of *CLDN11* was significantly more frequent in skin metastases (79%) compared to brain metastases (31%; *p =* 0.007). *CLDN11* methylation was also found in five out of six MM cell lines (83%) and its promoter hypermethylation correlated with a reduced expression. Treatment of MM cell lines with a DNA methylation inhibitor reactivated *CLDN11* transcription by its promoter demethylation. In summary, *CLDN11* proved to be an epigenetically inactivated tumor related gene in melanomagenesis, and analysis of *CLDN11* methylation level represents a potential tool for assisting in the discrimination between malignant melanoma and nevus cell nevi.

## 1. Introduction

**Figure 1:**
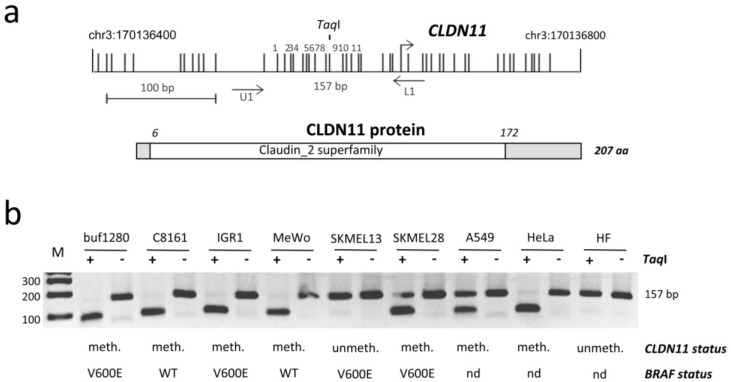
Epigenetic regulation of *Claudin 11* (*CLDN11*) in malignant melanoma. (**a**) Structure of the *CLDN11* CpG island promoter on chromosome 3 and the CLDN11 protein (207 aa). Arrows mark transcriptional (+1) start site for *CLDN11.* Vertical lines indicate CpGs. The 157 bp PCR product with respective primers and the *Taq*I site are depicted. The conserved Claudin_2 superfamily domain of CLDN11 is marked; (**b**) Methylation analysis of *CLDN11* by COBRA. Bisulfite-treated DNA from MM cell lines (buf1280, C8161, IGR1, MeWo, SKMEL13 and SKMEL28), lung cancer A549, HeLa and human fibroblasts (HF) was amplified, digested with *Taq*I (+) or mock digested (−) and resolved on 2% gels with a 100 bp marker (M). The methylation status of *CLDN11* (meth./methylated and unmeth./unmethylated) and BRAF status (WT/wild type, V600E/Codon 600 mutation and nd/not determined) are indicated.

Malignant melanoma is a malignant skin cancer showing a rising incidence worldwide [1]. Several molecular pathways have been found altered in melanocytic tumors including the MAPK pathway, the p16^INK4a^/RB pathway and the Hippo/Ras Association Domain Family (RASSF) pathway [2,3,4]. Aberrant regulation of these pathways is accomplished through inactivation of tumor suppressor genes (e.g., *RASSF10*) and activation of proto-oncogenes (e.g., *BRAF*) [5,6]. Apart from mutation, the epigenetic silencing of tumor suppressor genes is a frequent and fundamental event in the pathogenesis of cancer, including melanomagenesis [7,8]. This inactivation is achieved by hypermethylation of CpG island promoters in malignant melanoma. In this context, methylation markers may serve as important tools to distinguish between benign lesions and aggressive tumors. Recently, it has been suggested that *Claudin 11* (*CLDN11*) could be a useful epigenetic biomarker for identifying malignant melanoma [9]. *CLDN11* is a member of the claudin family that encodes integral membrane proteins and is involved in the formation of the paracellular tight junction seal in tissues [10,11]. Thus *CLDN11* harbors a Claudin_2/PMP22 domain (Figure 1a) that is also found in the peripheral myelin protein PMP22 and the epithelial membrane proteins (e.g., EMP1) [12]. So far, 27 members of the *CLDN* family (*CLDN1* to *27*) have been identified in the human genome [13]. Expressional analysis suggests that several claudin genes exhibit decreased transcript levels in cancer. However, CLDN3, CLDN4 and CLDN7 levels are elevated in certain tumor entities [10]. For *CLDN11* it has been reported that it is silenced in gastric cancer by promoter hypermethylation and its inactivation is associated with invasiveness of this cancer [14]. A genome-wide analysis has identified the methylation of *CLDN11* in primary cutaneous melanoma [15]. However, the epigenetic regulation (e.g., expression) in melanoma has not been analyzed.

The aim of our study was to illuminate the epigenetic inactivation of *CLDN11* in malignant melanomas (MM) in more detail. Here, we report a significant increase in the methylation level of *CLDN11* in MM metastases compared to primary MM and nevus cell nevi.

## 2. Results

### 2.1. Epigenetic Inactivation of CLDN11 in Malignant Melanoma

Recently hypermethylation of *Claudin 11* (*CLDN11*) has been reported in primary melanomas [9], however its epigenetic regulation was not analyzed in detail. The schematic promoter region of *CLDN11* and according CpGs are shown in Figure 1a. The promoter lies within a CpG island of 1644 bp on chromosome 3q26.2 from position 170′136′243 to 170′137′886 (UCSC genome browser). To reveal the epigenetic status of *CLDN11* in malignant melanoma (MM) cell lines, we have analyzed its aberrant methylation in buf1280, C8161, IGR1, MeWo, SKMEL13, SKMEL28, lung cancer (A549), cervix cancer (HeLa) and human fibroblast (HF) by COBRA (Figure 1b). Fragmentation of the PCR product by *TaqI* indicates an underlying methylated *CLDN11* promoter. In five MM cell lines (buf1280, C8161, IGR1, MeWo, SKMEL28) hypermethylation of *CLDN11* was detected (Figure 1b). *CLDN11* was unmethylated in normal human fibroblast (HF) and melanoma cell line SKMEL13. Methylation of *CLDN11* was also observed in A549 and HeLa cancer cells (Figure 1b). Previously, we analyzed the BRAF mutational status in MM cell lines [5]. There was no obvious correlation between *CLDN11* methylation and BRAF mutation status in MM cell lines (Figure 1b). 

**Figure 2:**
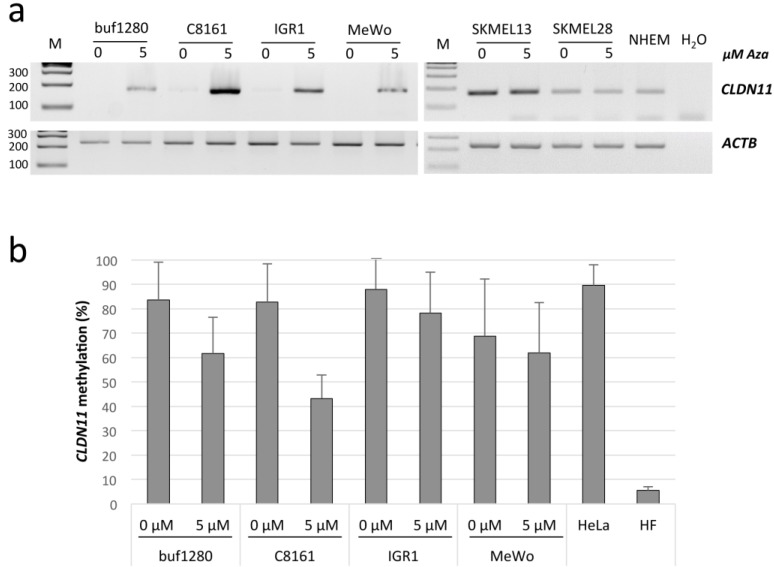
Epigenetic reactivation of *CLDN11* in malignant melanoma. (**a**) RNA expression of *CLDN11* in MM cell lines and normal human epidermal melanocytes (NHEM). MM cell lines (buf1280, C8161, IGR1, MeWo, SKMEL13 and SKMEL28) were treated for four days with 5 µM of 5-aza-2′-deoxycytidine (Aza). RNA was isolated and analyzed by RT-PCR. Products for *CLDN11* (167 bp) and a 100 bp ladder (M) were resolved on 2% gel. Expression of *ACTB* (225 bp) was determined as a control for RNA integrity; (**b**) Methylation of *CLDN11* in Aza-treated MM cell lines. DNA was isolated and analyzed by quantitative bisulfite pyrosequencing. 11 CpGs within the PCR products obtained from the indicated MM cell lines, HeLa and human fibroblasts (HF) were analyzed. The mean frequency of CpG methylation is indicated.

Subsequently, we analyzed the expression of *CLDN11* in six MM cell lines and normal human epidermal melanocytes (NHEM) by RT-PCR (Figure 2a). *CLDN11* mRNA levels were reduced in buf1280, C8161, IGR1 and MeWo compared to NHEM (Figure 2a). Treatment of these four cell lines with 5-aza-2′-deoxycytidine (Aza), a substance that inhibits DNA methylation, resulted in increased *CLDN11* expression (Figure 2a). In SKMEL13 cells, which harbor an unmethylated promoter, *CLDN11* expression was observerd in untreated cells (Figure 2a). In SKMEL28 cells with a partially methylated *CLDN11* promoter, there was no induction of *CLDN11* expression after Aza treatment. To analyze the impact of Aza treatment on DNA methylation, quantitative bisulfite sequencing was performed (Figure 2b). For all four MM cell lines that exhibited elevated *CLDN11* expression after Aza treatment, a demethylation of *CLDN11* was detected (Figure 2). Especially in C8161 which exhibit a high re-expression of *CLDN11* (Figure 2a), a strong demethylation (2-fold reduction in methylation level) of the *CLDN11* promoter region was observed after Aza treatment (Figure 2b).

### 2.2. CLDN11 Promoter Hypermethylation Occurs Frequently in Melanoma, Is a Rare Event in Nevi

**Figure 3:**
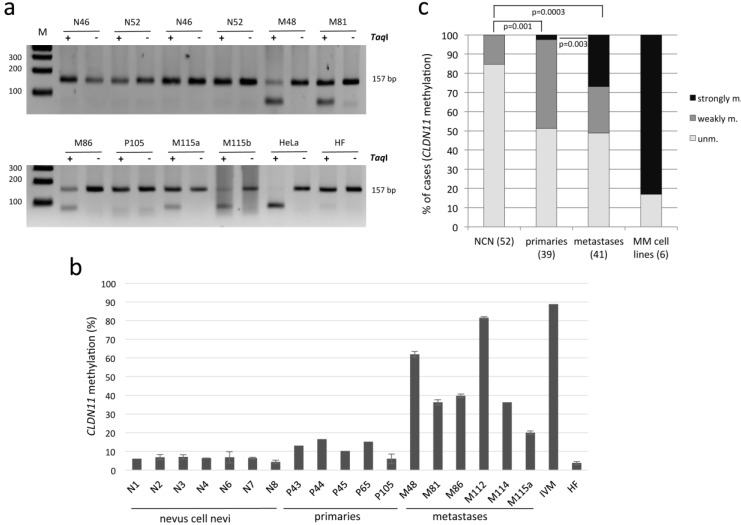
Methylation of *CLDN11* in nevus cell nevi (N), primary malignant melanoma (P) and melanoma metastases (M). (**a**) Methylation of *CLDN11* was analyzed by COBRA. PCR products (157 bp) from bisulfite-treated DNA were digested with *TaqI* (+) or mock digested (−) and resolved on 2% gel. DNA from HeLa and fibroblast (HF) was utilized as methylated and unmethylated control. Sizes of a 100 bp ladder (M) are marked; (**b**) Methylation analysis of *CLDN11* by bisulfite pyrosequencing. 11 CpGs within PCR products generated from nevus cell nevi (N), primary MM (P/primaries) and MM metastases (M) and *in vitro* methylated DNA (IVM) were analyzed in technical replicates. M48 is a lymph node metastasis and all other metastases originated from skin; (**c**) Comparison between methylation of MM (primaries, metastases) and NCN. Bar charts indicate percentage of cases with unmethylated (unm. <10%), weakly methylated (10% to 20%) and strongly methylated (>20%) *CLDN11*. Significance was calculated with the two-tailed Fisher exact probability test.

Subsequently, we analyzed the methylation of *CLDN11* in primary MM, MM metastasis and NCN by COBRA and bisulfite pyrosequencing (Figure 3 and Table 1). In 21 out of 41 (51%) metastases (e.g., M48/lymph node and M81/skin metastasis), the promoter of *CLDN11* was methylated and therefore restriction products were detected (Figure 3a and Table 1). Methylation of *CLDN11* was frequently found in 15 out of 19 (79%) skin metastases of MM, but only in five out of 16 (31%) brain metastases of MM (*p =* 0.007; Table 1). Methylation levels of 11 CpGs (Figure 1a) were quantified by pyrosequencing and rated unmethylated (<10%), weakly methylated (10% to 20% methylation) and strongly methylated (>20%; Figure 3b,c). The background was under 10% in all samples that were negative in COBRA (Figure 3 and data not shown). None of the 52 (0%) NCN exhibited strong methylation of *CLDN11* and only 15% (8/52) NCN showed weak *CLDN11* methylation. In primary melanoma a significantly higher frequency of *CLDN11* methylation (49%; 19/39) was revealed (*p =* 0.001). Interestingly, primary melanoma exhibited lower methylation levels compared to metastases (Table 1, Figure 3b,c). We observed an increase in methylation levels with significantly stronger methylated metastases compared to primaries (27% *vs.* 3%; *p =* 0.003, respectively; Figure 3c and Table 1). In summary, our data suggest that hypermethylation of *CLDN11* occurs frequently in primary MM and metastases, however is rarely found in NCN (*p =* 0.001 and *p =* 0.0003, respectively).

**Table 1:** Summary of *CLDN11* methylation (*n*).

Case	Unmethylated ^a^	Weakly Methylated ^b^	Strongly Methylated ^c^
**Nevus cell nevus**			
dysplastic (14)	86% (12)	14% (2)	0%
Non-dysplastic (38)	84% (32)	16% (6)	0%
Total (52)	85% (44)	15% (8)	0%
**Primary melanoma (39)**	51% (20)	46% (18)	3% (1)
**Melanoma metastasis**			
Skin (19)	21% (4)	37% (7)	42% (8)
Brain (16)	68.75% (11)	18.75% (3)	12.5% (2)
Lymph node (4)	75% (3)	0%	25% (1)
Others (2)	100% (2)	0%	0%
Total (41)	49% (20)	24% (10)	27% (11)
**MM cell lines (6)**	17% (1)	0%	83% (5)

^a^ <10%; ^b^ 10%–20%; ^c^ >20% methylation.

## 3. Discussion

The *Claudin* gene family consists of 27 members, which encode membrane proteins of the paracellular tight junction. *Claudin 11* (*CLDN11*) was recently identified as a member that is hypermethylated in human cancers including malignant melanoma (MM) [9,14]. Here we confirm that *CLDN11* is frequently hypermethylated in cutaneous MM, including primaries and metastases (49% and 51% methylation, respectively). Additionally to previous studies, we have performed quantitative methylation analysis and revealed significantly increased methylation levels of MM metastases especially in skin metastases (Figure 3 and Table 1). Moreover, we show that hypermethylation of *CLDN11* correlates with inactivation of its expression and that inhibition of DNA methyltransferase epigenetically reactivated *CLDN11* transcription (Figure 2). Gao *et al.* have reported a similar frequency of 48% methylation for primary melanoma, but 73% for metastatic melanoma [9]. However, we show that the location of metastases correlates significantly with the methylation frequency of *CLDN11* (Table 1). Especially skin metastases exhibit significantly higher methylation frequency compared to brain metastases (79% compared to 31%, respectively). It is tempting to speculate that *CLDN11* methylation levels in primary MM contribute to differences in metastatic capacity of melanomas. Thus it will be interesting to analyze the functional consequences of *CLDN11* inactivation for invasiveness potential of melanomas in more detail. Interestingly, the RASSF6 tumor suppressor gene exhibited its highest methylation frequency in melanoma brain metastases [16]. Since most melanomas are driven by an activating mutation at codon 600 of BRAF, we also analyzed its mutational status and *CLDN11* methylation in MM cell lines (Figure 1b). There was no direct or inverse correlation between both events. It has been reported that BRAF mutations are found with similar frequencies in brain metastases (48%) and skin metastases (53%) [17]. We revealed 66% of BRAF mutation and 83% of *CLDN11* methylation in MM cell lines (Figure 1b). To date, the methylation rate of *CLDN11* had not been analyzed in MM cell lines.

Moreover, we utilized bisulfite pyrosequencing, a method that provides quantitative data on the methylation levels of *CLDN11* in different skin samples. Here we report significantly elevated methylation levels in metastatic MM compared to primary cancers (Figure 3c), thus the frequency and level of *CLDN11* methylation increases with the malignancy of melanoma. Previously, we have analyzed *RASSF10* methylation in MM and we reported frequent methylation in melanoma, although *RASSF10* methylation was not found in non-dysplastic nevi [6]. Here, we observed a weak *CLDN11* methylation in few non-dysplastic and at a similar frequency (15%) in dysplastic NCN (Table 1). Gao *et al.* reported only 3% methylation of *CLDN11* methylation in dysplastic NCN [9]. However they have utilized methylation specific PCR and analyzed a region within the 1st Exon (+100 to +300) [9]. Since we have analyzed a region upstream of the transcriptional start site (Figure 1a), the difference in methylation could be attributed to gradual spreading of DNA methylation from the borders of the *CLDN11* CpG island. This progressive epigenetic inactivation event has been reported previously for human mammary epithelial cells during stress induced senescence for RASSF1A [18]. Considering DNA methylation as an epigenetic biomarker for MM the region of *CLDN11* where methylation occurs during melanomagenesis should be utilized, since low methylation levels were also observed in NCN.

Additionally, we have also analyzed the epigenetic regulation of *CLDN11* in MM cell lines (Figure 1). The level and frequency of *CLDN11* hypermethylation is increased compared to primary tissues (Figure 3c). Moreover, we found that aberrant *CLDN11* promoter methylation correlates with its transcriptional silencing in four MM cell lines (Figure 2). Inhibition of DNA methyltransferases by 5-aza-2′-deoxycytidine reactivates *CLND11* expression through promoter demethylation in these cell lines. These observations suggest that aberrant DNA methylation has an important impact on *CLDN11* expression, which had not been analyzed previously in MM.

Aberrant epigenetic regulation of other *Claudin* members has also frequently been reported in human cancers. *CLDN1* methylation was detected in colon cancer and has been found in breast cancer [19,20]. For *CLDN3* it has been observed that it methylation occurs in esophageal and hepatocellular carcinoma [21,22]. Methylation of *CLDN4* and *CLDN5* has been reported in bladder and pancreatic cancer, respectively [23,24]. Moreover, hypermethylation of *CLDN6*, *CLND7* and *CLDN15* have also been observed in different cancer entities [25,26,27]. Thus hypermethylation of distinct *Claudins* were frequently reported in human cancers and it will be interesting to analyze the methylation of several *Claudin* family members (e.g., *CLDN1* and *CLDN15*) in cutaneous melanoma.

## 4. Experimental Section 

### 4.1. Tissue and Cell Lines 

Primary tissues and cancer cell lines were previously published [6]. All patients signed informed consent at initial clinical investigation. The study was approved by local ethic committees (University of Heidelberg, Heidelberg, Germany). Primary Normal Human Epidermal Melanocytes (NHEM) obtained from PromoCell (Heidelberg, Germany). All cell lines were cultured in humidified atmosphere (37 °C) with 5% CO_2_ and 1xPenicillin/Streptomycin in according medium. 

### 4.2. Methylation Analysis 

DNA was isolated by phenol-chloroform extraction and then bisulfite treated prior to COBRA analysis and pyrosequencing [28]. Methylation analyses were performed in technical replicates. Bisulfite treated DNA (150 ng) was used for PCR with primer CLDN11BSU1 (TTTTGGGGTTATTTTGTTTTTTTTTA) and 5′-biotinylated primer CLDN11BioL1 (AAAACAACAACRCTACTAAACAAC). Products were digested with 0.5 µL *Taq*I (Fermentas GmbH, St. Leon-Rot, Germany) for 1 h at 65 °C and resolved on 2% TBE gel. Methylation status was quantified utilizing the primer CLDN11Seq1 (ATTTTGTTTTTTTTTAYGTTTTTTTT) and PyroMark Q24 (Qiagen, Hilden, Germany). Eleven CpGs are included in the analyzed region of *CLDN11* and mean methylation was calculated (Figure 1a). For *in vitro* methylation of genomic DNA we used CpG methyltransferase (M. SssI, NEB, Frankfurt, Germany).

### 4.3. Expression Analysis

RNA was isolated using the Isol-RNA lysis procedure (5 Prime, Hamburg, Germany). RNA was DNase (Fermentas GmbH, St. Leon-Rot, Germany) digested and then reversely transcribed [29]. RT-PCR was performed with primers: CLDN11RTF2: CCCACCTGCCGCAAGCTGGA, CLDN11RTR2: GGCAGACCCAGGACCGAGGC, ßACTFW: CCTTCCTTCCTGGGCATGGAGTC, ßACTRW: CGGAGTACTTGCGCTCAGGAGGA.

### 4.4. Statistical Evaluation

Categorical variables were plotted into contingency tables and evaluated using Fisher’s exact probability test. All reported *p*-values are two-sided and considered significant for *p <* 0.05.

## 5. Conclusions

In summary, our results show that hypermethylation of *CLDN11* promoter occurs frequently in MM, but was rarely found in NCN. This data suggests that *CLDN11* may encode a novel melanoma-specific tumor suppressor gene. Further studies are necessary to elaborate the exact tumor suppressor function of CLDN11. Furthermore, quantitative *CLDN11* methylation analysis may serve as candidate biomarker tool in melanomagenesis in combination with other markers (e.g., *RASSF10*), since low *CLDN11* methylation levels were also observed in NCN.

## Abbreviations

CLDN11: Claudin 11
COBRA: combined bisulfite restriction analysis
Aza: 5-Aza-2′-deoxycytidine
MM: malignant melanoma
NCN: nevus cell nevus
RASSF: Ras Association Domain Family
ACTB: Beta-Actin
